# Identification of minimum essential therapeutic mixtures from cannabis plant extracts by screening in cell and animal models of Parkinson’s disease

**DOI:** 10.3389/fphar.2022.907579

**Published:** 2022-10-05

**Authors:** Michael G. Morash, Jessica Nixon, Lori M. N. Shimoda, Helen Turner, Alexander J. Stokes, Andrea L. Small-Howard, Lee D. Ellis

**Affiliations:** ^1^ National Research Council of Canada, Halifax, NS, Canada; ^2^ Laboratory of Immunology and Signal Transduction, School of Natural Sciences and Mathematics, Chaminade University, Honolulu, HI, United States; ^3^ Laboratory of Experimental Medicine, John A Burns School of Medicine, University of Hawaii, Honolulu, HI, United States; ^4^ GBS Global Biopharma, Inc., Ottawa, ON, Canada

**Keywords:** Parkinson’s disease, cannabinoids, zebrafish, dopamine, neuroprotection, movement disorder, cannabis, cannabidiol

## Abstract

Medicinal cannabis has shown promise for the symptomatic treatment of Parkinson’s disease (PD), but patient exposure to whole plant mixtures may be undesirable due to concerns around safety, consistency, regulatory issues, and psychoactivity. Identification of a subset of components responsible for the potential therapeutic effects within cannabis represents a direct path forward for the generation of anti-PD drugs. Using an *in silico* database, literature reviews, and cell based assays, GB Sciences previously identified and patented a subset of five cannabinoids and five terpenes that could potentially recapitulate the anti-PD attributes of cannabis. While this work represents a critical step towards harnessing the anti-PD capabilities of cannabis, polypharmaceutical drugs of this complexity may not be feasible as therapeutics. In this paper, we utilize a reductionist approach to identify minimal essential mixtures (MEMs) of these components that are amenable to pharmacological formulation. In the first phase, cell-based models revealed that the cannabinoids had the most significant positive effects on neuroprotection and dopamine secretion. We then evaluated the ability of combinations of these cannabinoids to ameliorate a 6-hydroxydopmamine (OHDA)-induced change in locomotion in larval zebrafish, which has become a well-established PD disease model. Equimolar mixtures that each contained three cannabinoids were able to significantly reverse the OHDA mediated changes in locomotion and other advanced metrics of behavior. Additional screening of sixty-three variations of the original cannabinoid mixtures identified five highly efficacious mixtures that outperformed the original equimolar cannabinoid MEMs and represent the most attractive candidates for therapeutic development. This work highlights the strength of the reductionist approach for the development of ratio-controlled, cannabis mixture-based therapeutics for the treatment of Parkinson’s disease.

## Introduction

Neurodegeneration in Parkinson’s disease (PD), Alzheimer’s disease, Lewy Body Dementia, and Huntington’s disease is a growing health burden. Among these, the pathophysiology of PD has been intensively studied, but its underlying cause remains enigmatic (Gan-Or et al., 2015; Bandres-Ciga et al., 2020; Mani et al., 2021). Mechanistically, motor symptoms of PD are linked to the death of dopamine (DA)-producing neurons in the substantia nigra (Surmeier, 2018; Gonzalez-Rodriguez et al., 2020) and to the deposition of misfolded alpha-synuclein protein aggregates in Lewy bodies (Greenamyre and Hastings, 2004; Lin et al., 2019). Desensitization of the DA response system has also been documented, suggesting that both DA production and efficacy are compromised in PD (More and Choi, 2015). Most of the agents currently approved for treating PD address symptoms of DA depletion, such as bradykinesia, and do not modify disease progression. Levodopa remains the most common symptomatic treatment for PD; however, 30–35% of patients develop Levodopa Induced Dyskinesia (LID) after as little as 24 months of Levodopa usage (Utsumi et al., 2013; More and Choi, 2015). Given these significant side effects, there remains a need for non-Levodopa based symptomatic therapies for PD.

The potential for cannabis-derived compounds to provide symptom improvement in PD patients is suggested by anecdotal and patient reported outcomes (PRO) data (Venderova et al., 2004; Feeney et al., 2021; Yenilmez et al., 2021). Unfortunately, with native cannabis or cannabis extracts, there are unnecessary and unwanted psychoactive side effects from delta-9 tetrahydrocannabinol (THC) (Oultram et al., 2021), along with plant material contamination and complexities in the accurate delivery of therapeutic extracts (Couch et al., 2020; Wiseman et al., 2021) that may compromise patient safety. Additionally, single cannabinoid therapeutics composed of THC or cannabidiol (CBD) do not fully recapitulate the PRO effectiveness of the native plant (Turner et al., 2017) or it’s extracts (Blasco-Benito et al., 2018; Russo, 2018; Ferber et al., 2020). This suggests that cannabis plant extracts, which contain hundreds of compounds, include components other than these major cannabinoids that contribute significantly to their effectiveness. The pharmacodynamic properties of the cannabinoid and terpene active ingredients from cannabis plant extracts have been described in detail (Maayah et al., 2020; Patricio et al., 2020). In the cannabinoid research field, the ability of cannabis-derived ingredients to act synergistically by enhancing or diminishing the net effectiveness of a therapy has been identified and is referred to as the ‘entourage effect’ (Ben-Shabat et al., 1998; Russo, 2011). While the entourage effect is typically expected to be modulated pharmacodynamically through the interactions of multiple ligands with one or more receptors, pharmacokinetic effects such as metabolism have also been demonstrated (Cogan, 2020). This makes assessing their activity more complicated, but also makes them potentially more effective therapeutics than single target drugs due to positive co-operative interactions. The cannabinoids and terpenes from cannabis plant extracts are ligands of multiple receptors including metabotropic cannabinoid receptors, ionotropic cannabinoid receptors, serotonin receptors, and orphan G-protein coupled receptors, making it likely that they would individually act as multi-target drugs (Stasilowicz et al., 2021), and indeed cannabis extracts demonstrate more potency than CBD alone in cell based assays (Milligan et al., 2022). A number of minor cannabinoids have been shown to bind to CB1 and CB2 receptors, although with varying affinities (Zagzoog et al., 2020). While some terpenes have shown an additive effect with cannabinoid agonists in rodents (LaVigne et al., 2021), the direct interaction of terpenes with the CB receptors (Santiago et al., 2019; Finlay et al., 2020) and TRP channels (Heblinski et al., 2020) is contested. Developing a model to study these complex interactions is a critical step to the rational design of multi-component, efficacious, cannabis-inspired therapeutics for PD.

Based on the complexity, side effects, and off target interactions that may be inevitable using whole plant extracts, it is essential to identify the core components of cannabis that are required for the treatment of PD. To this end in 2016, using a combination of *in silico* (Reimann-Philipp et al., 2020) and cell based assays, GB Sciences identified and patented a mixture of 8 essential cannabis components that when combined with CBD or cannabinol (CBN) recapitulated the anti-Parkinsonian activity anecdotally ascribed to whole plant cannabis (U.S. Patent Number 10,653,640). These compounds include the three minor cannabinoids cannabigerol (CBG), cannabichromene (CBC), and cannabidivarin (CBDV) (<5% of the original cannabis extracts), and five terpenes (α-pinene, trans-nerolidol, limonene, linalool, and phytol). While the identification of 8 compounds from a pool of >100,000 represents a tremendous reduction in complexity, unfortunately these mixtures remain difficult to formulate into therapeutics owing to the diversity of the chemical structures and the differences in pharmacokinetics of each component. Aside from the practical difficulties in creating therapeutics containing multiple drugs, known as Fixed-dose Drug Combinations (FDCs), these polypharmaceuticals also present challenges with respect to patient interactions (Gautam and Saha, 2008). FDCs can pose a challenge with dosage adjustments of individual drugs, drug interactions, and off-target effects, with each additional ingredient creating more opportunities for adverse reactions. Thus, the identification of the minimal set of cannabis ingredients that can recapitulate the effects of whole plant is crucial in the creation of a multicomponent therapeutic.

Therefore in this study we sought to further reduce the number of compounds in the patented formulation to a minimal essential mixture (MEM) that could recapitulate as many of the effects of the original combination as possible with the goal of generating a mixture that would be more amenable to pharmacological production. Two cell assays were initially used to evaluate the potential therapeutic efficacy of the mixtures by using both an *in vitro* neuroprotection assay and a dopamine secretion assay in dopaminergic neuronal cell models. From these cell assays, we identified the cannabinoids as being largely responsible for the activity seen in the patented mixture with a nominal effect of the terpenes. We then assayed multiple drug combinations that contained three individual cannabinoids for their ability to ameliorate a 6-hydroxydopmamine (OHDA)-induced model of PD in zebrafish larvae. The results have allowed us to move sequentially from the remarkable chemical complexity of the cannabis plant, to moderately complex mixtures with potential PD-therapeutic activity as evaluated in cell models, to refined minimal essential mixtures of cannabinoids that demonstrate therapeutic effects on OHDA treated zebrafish. The sequentially reductionist process used in this study preserves some of the entourage-like effects of whole plant extracts, while achieving ‘relative’ simplicity within MEM that is a requirement for obtaining the manufacturing and quality control advantages of single ingredient drugs.

## Materials and methods

### Chemicals and cell lines

All cannabinoids used in this study were purchased as 1 mg/ml standards in methanol (Sigma, Ontario, Canada): Cannabidiol (CBD), Cannabichromene (CBC), Cannabidivarin (CBDV), Cannabigerol (CBG), and Cannabinol (CBN). The α-pinene (98% purity), trans-nerolidol (>85% purity), and Methanol (99.9% purity) were also purchased from Sigma (Sigma, Ontario, Canada). D-Limonene (96.9% purity) was purchased from MPBIO (MP Biomedicals LLC, Ohio, USA), Linalool (>96% purity) was purchased from TCI (TCI, Oregon, USA) and Phytol was from Agilent Technologies (Agilent Technologies, Inc., Rhode Island, USA). All terpenes were diluted in methanol. 6-hydroxydopamine (OHDA) (Sigma, Ontario, Canada) was diluted in saline buffer (0.9% NaCl) supplemented with 0.02% Ascorbic acid. Mixtures produced for cell line experiments used equimolar components as follows: MIX-1 = minor cannabinoids (CBC, CBG and CBDV) + Terpenes (Linalool, α-pinene, limonene, t-nerolidol and phytol), MIX-2 = terpenes only, and MIX-3 = Minor cannabinoids only. Individual major cannabinoids, CBD and CBN, were added at the same equimolar amount. Please refer to legends in Figures 3, 6 for specifics regarding mixture compositions.

Cell lines used were from ATCC (Manassas, Virginia, United States). Cath.a cells (sp. = mouse, cat# CRL-11179), a CNS catecholaminergic cell line, were cultured according to ATCC instructions and were induced to CAD differentiated status by serum deprivation (0.5% FBS culture for 36 h) prior to experiments as described (Qi et al., 1997). PC12 cells (sp. = rat, ATCC #CRL-1721) were cultured in RPMI 10% FBS. PC12 differentiation used Minimal Essential Medium containing 1% HS and 0.5% FBS, then the cells were treated with 100 ng/ml NGF, 100 ng/ml basic fibroblast growth factor (bFGF), and serum-starved media containing 2 mg/ml BSA for 2 days (Jeon et al., 2010). Schematic representations of exposure paradigms available in Supplementary Figure S2.

### *In vitro* neuroprotection assays

**FIGURE 1:**
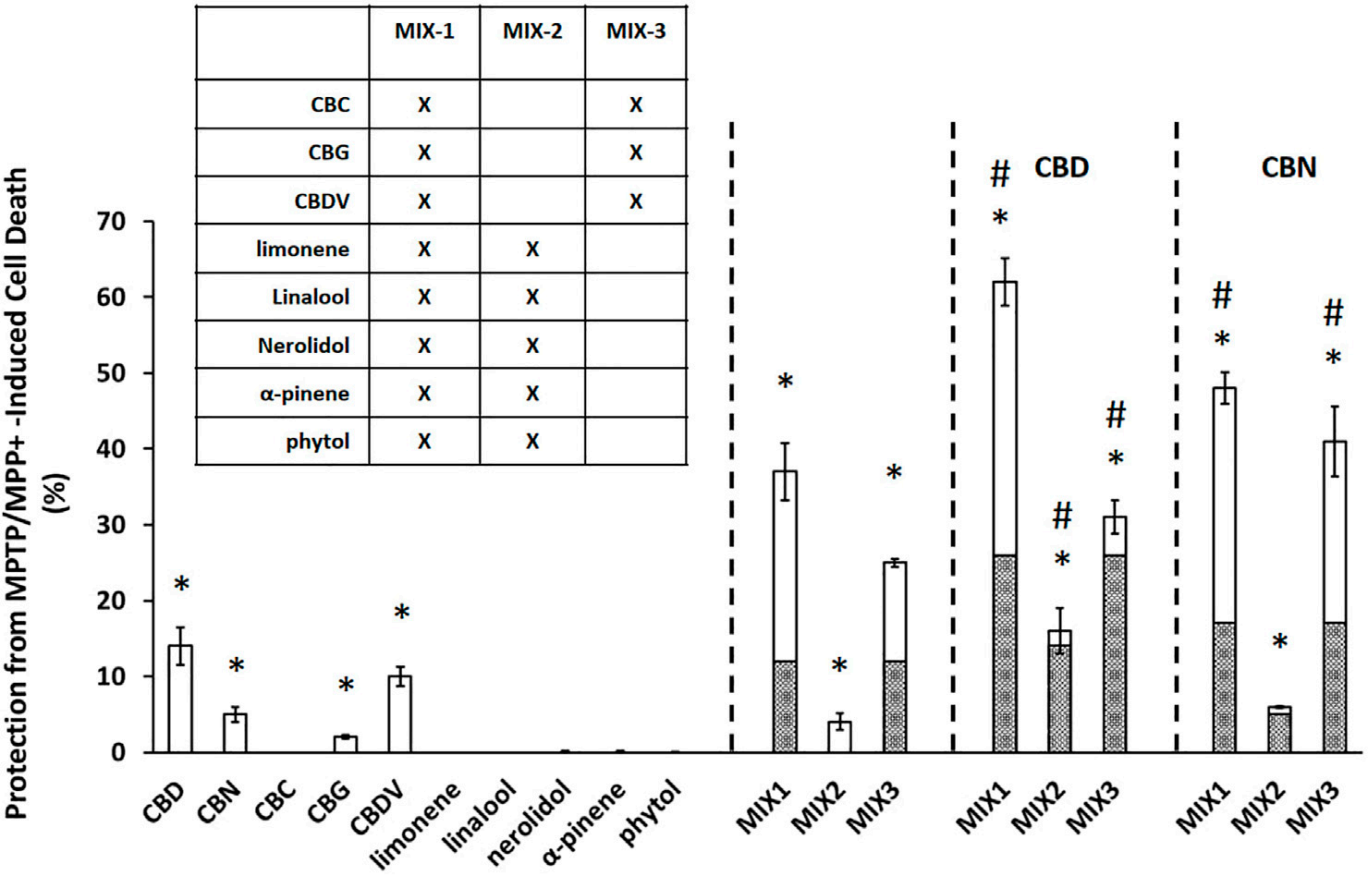
Cannabinoids produce significant neuroprotection in MPTP/MPP^+^ assay. Data are presented as the percent protection from MPTP/MPP^+^ cell death evaluated based on the MTT cell viability assay, where the experimental value is normalized relative to the vehicle control. An asterisk * indicates a *p*-value <0.05 for the replicates relative to their respective vehicle control replicates. MIX-1, MIX-2, and MIX-3 tested without or with the addition of a major cannabinoid (CBD or CBN) a hashtag # represents *p* < 0.05 major cannabinoid vs. native mixture. Calculated (hashed shading) prediction of efficacy based on the sum of the efficacy of each ingredient measured separately and Measured (open shading) efficacy are shown. Each data point in the figure represents the mean ± the standard deviation of twenty-four experimental results obtained at 10 µM of each major or minor cannabinoid and terpene (alone or in equimolar mixtures as described in the inset table in Panel). Twenty-four experimental results were obtained by repeating eight independent experiments three times on three different days (”8 × 3”).

Neuroprotective effects were assessed based on the ability of both individual compounds and mixtures of compounds to protect against neuronal cell death induced by 1-methyl-4-phenylpyridinium (MPP^+^) in the 1-methyl-4-phenyl-1,2,3,6-tetrahydropyridine (MPTP)-based selective cytotoxicity assay (Arshad et al., 2014). MPP^+^ is an active metabolite of MPTP that is known to cause human Parkinsonism after injection (Langston and Palfreman, 2014). As in Arshad et al. (2014), MPTP/MPP^+^ assays were performed *in vitro* on Cath. a cells by applying each compound or mixture of compounds to the cell cultures 18 h after application of MPP^+^ (Arshad et al., 2014). Cell viability assessments were performed using a standard MTT cell viability assay (MTT Cell Proliferation Assay Kit, Cayman Chemicals, Ann Arbor, Michigan, Item No. 10009365). Cell viability was assessed 24 h after exposure to MPP^+^, which is 6 h after exposure to the tested compound or compound mixture. Percent protection was normalized to MPTP control alone (100% cell death). To establish an effective dose range across the compounds in this assay system, the neuroprotective effects of each individual compound were tested at 5 different concentrations (Supplementary Figure S1). Based on these results the individual and equimolar mixtures were tested in the cell assays at 10 µM each (Figure 1). Equimolar mixtures contained 10 µM of each compound. In all cases vehicle controls contained methanol at equal concentrations to those found in test compounds/mixtures, ≤ 5%.

### *In vitro* dopamine-release assay

In parallel with the neuroprotection assays, cannabinoid and terpene compounds were tested alone and in mixtures to determine their effects on dopamine release from differentiated PC12 cells (Greene and Tischler, 1976). PC12 cells were differentiated as described (Jeon et al., 2010; Hu et al., 2018). Supernatant samples were collected from 3 replicate wells 30 min after application of PMA/Ionomycin (positive control) or the indicated compounds, and dopamine was measured in the medium using the Dopamine ELISA Kit #KA1887 from Abnova (Abnova, California, US) according to manufacturer’s instructions. For dopamine secretion, dopamine release was normalized to PMA/ionomycin control (0% baseline).

### Zebrafish

The fish used in this study were wild-type AB/Tubingen hybrids. Age-matched embryos were reared in Pentair Aquatic Ecosystem (Apopka, Florida, USA) nursery baskets (200 embryos per basket) on a ZebTec Recirculation Water Treatment System (Tecniplast, Buguggiate, Varese, Italy) at 28.5 ± 0.5°C, on a 14-h day–10-h night light cycle. All adult zebrafish husbandry and breeding was in accordance with the Canadian Council of Animal Care guidelines.

### Behavioral testing in zebrafish

All compounds were diluted in 100% methanol (MeOH) and experiments were performed in a HEPES buffered E3 (HE3) medium (5 mM NaCl, 0.17 mM KCl, 0.33 mM CaCl2-2H2O, 0.33 mM MgSO4-7H2O, 10 mM HEPES, pH 7.2). Individual 120 h post fertilization (hpf) larval zebrafish were transferred to a 48-well microtiter plate in 500 μL of HE3 media. Larvae were acclimated for at least 1 hour in a lighted 28.5°C incubator (photon flux: 3–5 μmol s−1 m^2^) prior to experimentation and larval behavior was analyzed using DanioVision larval tracking systems with EthoVisionXT14/15 software (Noldus Information Technology Inc., Virginia, USA). Distance traveled was measured using dynamic subtraction at 28.5°C over 120 min with the first 90 min under lighted conditions (15 µmol m-2 s-1), followed by alternating 5-min dark/light cycles. Each larvae represents an independent measurement. Any larvae that were dead or displayed phenotypic abnormalities were removed from analysis. 12 larvae were used in each experimental condition, and at least 2 replicates of each concentration were performed.

### 6-Hydroxydopamine Parkinson’s model development and advanced behavioral analysis

Larvae were exposed to varying concentrations of 6-hydroxydopamine (OHDA) from 48 to 120 h post fertilization (hpf). 15 dechorionated larvae were transferred in 5 ml of HE3 to each well of a six-well plate. The 5 ml exposure media was replaced daily, and ascorbic acid/saline buffer (used to resuspend OHDA) was used as a vehicle control. Larvae were then loaded into 48-well microtiter plate in 500 μL of HE3 media as described above. Schematic representations of exposure paradigms available in Supplementary Figure S2.

In addition to distance travelled, activity was measured as a percentage change in pixel density during data acquisition. The integrated visualization feature in EthoVision software (Noldus Information Technology Inc., Virginia, USA) was used to detect larval activity during three distinct activity types: high (greater than 0.5% pixel change per sampling), moderate (between 0.03 and 0.5%), and inactive (less than 0.03%) states. The frequency with which larvae switched between activity states and the cumulative duration of time spent in each activity state was then measured. Metrics were captured in 1 min bins, and the average over 90 min was used to calculate each metric in each activity state. For frequency calculations, the number of times larvae switched between activity states (high, moderate, and low) was calculated (termed Total Frequency). The cumulative amount of time spent in the high and moderate activity states combined (termed Cumulative Duration) was averaged over 90 min and measured as a percentage.

### Equimolar minimum essential mixture and defined cannabinoid-ratio MEM testing using OHDA PD model

Acute behavioral assays were performed on the cannabinoids selected for the study as described above except larvae were loaded into the well plates with 450 µL HE3. Immediately prior to recording 50 μL of 10 × test compound solution was added to each well. For the OHDA challenge experiments 150 µM OHDA was used for all challenge experiments. During media replacements at 72 and 96 hpf, 25 µL of 200X stock solutions of the respective cannabinoid or E-MEM was added to the exposure media. At 120 hpf larvae were washed with HE3 and transferred in 500 μL to 48-well plates. Behavioral analysis was then performed as described above. All experiments were performed at least in duplicate. The E-MEM selected for further study (above) were combined in non-equimolar ratios (defined cannabinoid-ratios DCR-MEM) and subjected to both the total distance and activity analysis metrics, as described above. This procedure was done in 3 steps, reducing a single component by 50% (a 1:2 ratio relative to their original equimolar concentrations) or 90% (a 1:10 ratio relative to their original equimolar concentrations), reducing 2 components by 50% (1:2) or 90% (1:10), or a defined cannabinoid-ratio (DCR)-MEM reducing 2 components by different cannabinoid-ratios (1:2 or 1:10 relative to their original equimolar concentrations).

### Analysis and statistical methods

The mean ± the standard deviations were calculated for all samples in the cell assays. A two-tailed student’s t-test was used to evaluate the statistical differences between sample types. Calculation of statistical significance for total distance traveled over 90 min was performed by one-way ANOVA using a Dunnett’s multiple comparison test using GraphPad Prism 7.04 software (La Jolla, California, United States). Comparisons of either OHDA + Drug vs. OHDA, or Drug vs. MeOH were performed as an unpaired Student’s t-test. An asterisk is used to represent a *p*-value less than 0.05 or lower, unless otherwise defined.

## Results

### Neuroprotective effects of cannabis-compounds alone and in combination

At 10 μM, all of the individual cannabinoids except CBC displayed some neuroprotective effects while none of the terpenes were able to prevent MPTP/MPP^+^ induced apoptosis (Figure 1). A mixture of the minor cannabinoids and the terpenes (MIX-1) showed a substantial neuroprotective effect with an increase in cell survival of 37 ± 3.8%. Mixtures of either the terpenes alone (MIX-2) or the minor cannabinoids (MIX-3) were then created to assess their contribution to the overall activity of MIX-1. MIX-2 (terpenes) showed a limited overall protection (4 ± 1.1%) while MIX-3 (minor cannabinoids) demonstrated similar activity to MIX-1 (25 ± 0.5%). The effects of the major cannabinoids were then assessed by adding each individually to the mixtures. CBD increased cell survival in all three mixtures (62 ± 3.1% vs. 37 ± 3.8% (MIX1), 16 ± 3% vs. 4 ± 1.1% (MIX-2) and 31 ± 2.2% vs. 25 ± 0.5% (MIX-3). The effects of the second major cannabinoid (CBN) were similar but less pronounced than those seen for CBD. Importantly, in all cases the mixtures were more neuroprotective than would have been estimated from the sum (hashed shading area of bars) of their individual effects.

### Effects of cannabis-compounds in mixtures on dopamine-release responses

**FIGURE 2:**
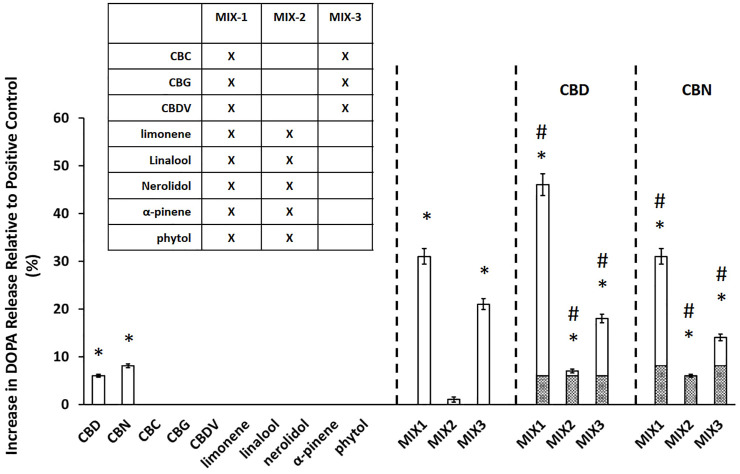
Cannabinoids mixtures elicit significant dopamine secretion. The experimental value is presented as the normalized value, which is a percent of the positive control value (secretion achieved with PMA/Ionomycin application). An asterisk * indicates a *p*-value <0.05 for the replicates relative to their respective vehicle control replicates. MIX-1, MIX-2, and MIX-3 were tested without or with the addition of a major cannabinoid (CBD or CBN). A hashtag # represents *p* < 0.05 major cannabinoid vs. native mixture. Calculated (hashed shading) prediction of efficacy based on the sum of the efficacy of each ingredient measured separately and Measured (open shading) efficacy were shown. Each data point in the figure represents the mean ± the standard deviation of twenty-four experimental results obtained at 10 µM of each major or minor cannabinoid and terpene (alone or in equimolar mixtures as described in the inset table in Panel B). Twenty-four experimental results were obtained by repeating eight independent experiments three times on three different days (”8 × 3”).

In parallel with the MPTP testing described in the previous section, we tested the effects of the same individual compounds and mixtures on dopamine release from PC12 cells (Figure 2). The major cannabinoids, CBD (6.0 ± 0.3% DOPA release) and CBN (8.1 ± 0.4% DOPA release), were the only individual compounds tested that produced a statistically significant (*p* < 0.05) increase in dopamine release. The relative performance of the mixtures in the dopamine assay was similar to the trend observed in the MPTP/MPP^+^ assay. MIX-1 led to the largest increase in DOPA release of 31 ± 1.6%, followed by MIX-3 (21 ± 1.1%), while the DOPA release for MIX-2 was not significant. Again, we also evaluated the effectiveness of adding the major cannabinoids (CBD or CBN) to each MIX relative to the effectiveness of each MIX alone. When CBD was added to MIX-1, the effectiveness was increased leading to a DOPA release of 46 ± 2.3% for MIX-1 + CBD**,** while adding CBN to MIX-1 did not significantly increase the DOPA release. The addition of CBD or CBN to MIX-2 were able to produce modest but significant increases in dopamine secretion up to 7.0 ± 0.4% DOPA release for MIX-2 + CBD and 6.0 ± 0.3% DOPA release for MIX-2 + CBN. Similarly, the addition of CBD to MIX-3 produced an increase in DOPA release relative to MIX-3 alone (MIX-3 + CBD produced 18 ± 0.9% DOPA release), while the addition of CBN to MIX-3 produced a significant reduction in the DOPA release compared to MIX-3 alone (MIX-3 + CBD produced 14 ± 0.7% DOPA release). As in the neuroprotection assays, mixtures of the components were able to elicit significantly more robust responses than the individual components.

Taken together, these results demonstrate that the effects of the mixtures cannot be attributed to a single ingredient. On the contrary, it suggests that interactions between the components in the mixtures are critical for the maximal efficacy of the mixture. In addition, it appears from the cell assay data that the terpene components of the mixtures have a minimal contribution on their efficacy and that the cannabinoid components are sufficient to use as a potential therapeutic.

### Assessment of cannabinoid effects in a zebrafish OHDA Parkinson’s model

**FIGURE 3:**
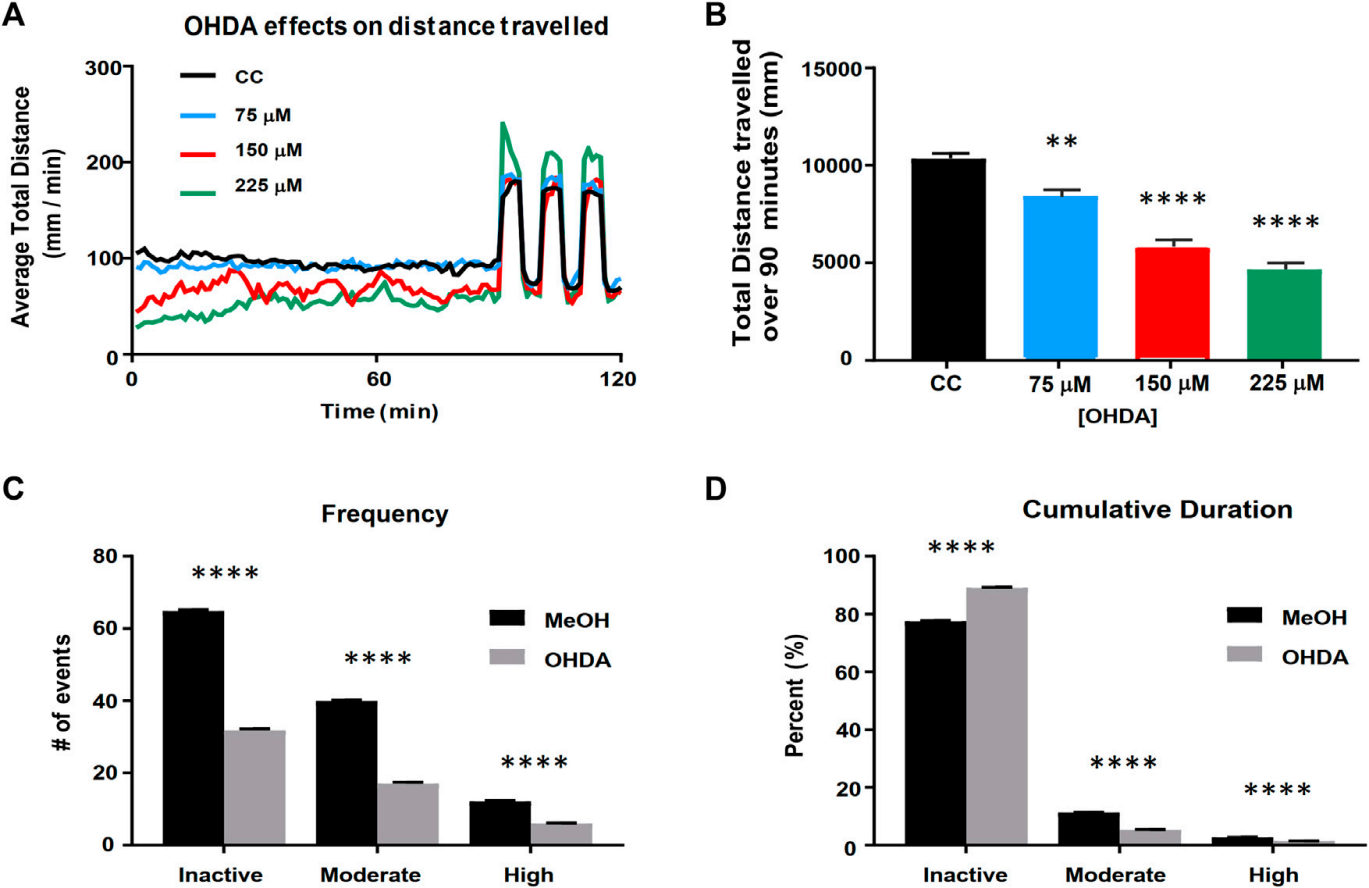
Validation of the larval zebrafish OHDA model. Panel **(A)** Behavioral profiles of total distance traveled (60 s bins) following OHDA exposure from 48 to 120 hpf. Panel **(B)** Total distance travelled during the first 90 min in the light following OHDA exposure from 48 to 120 hpf (n = 36). Advanced activity analytics **(C,D)** of Total Frequency of switching between activity states **(C)** and the nested Cumulative Duration in each activity state **(D)** (n = 48). CC vs. OHDA, ** = *p* < 0.01 **** = *p* < 0.0001.

In order to test whether the cannabinoids and cannabinoid-based mixtures identified by the cell screening assays would potentially alleviate symptomatic effects in an animal model of Parkinson’s disease, we applied a previously developed zebrafish larval model of dopamine cell loss caused by exposure to 6-hydroxydopamine (OHDA) (Feng et al., 2014; Cronin and Grealy, 2017; Benvenutti et al., 2018) and refined it based on a determination of OHDA dose and time conditions. We found that larval exposure to OHDA produced a concentration-dependent decrease in the baseline activity during an initial 90-min period in the light (Figures 3A,B). It also led to a non-significant increase in the maximum response to the light/dark transition (startle response) at 225 µM. Based on these findings, we selected 150 µM OHDA as our testing model as it produced a decrease in activity during the 90-min baseline period, which can be considered a model of bradykinesia, while not impacting the startle response which suggests a minimal effect on general locomotor function (Figure 3). Visual assessment of the OHDA treated larvae appeared to show a more complex pattern of behavior than could be assessed by a simple measure of distance travelled. We observed that OHDA treated larvae, when at rest, displayed a small, periodic side-to-side movement with no velocity that may represent a ‘resting tremor’. This behavior had not been previously defined and further highlights the significance of the OHDA exposure to act as a model of PD. Thus, in addition to analyzing distance travelled by the larvae as described above, larval activity was also analyzed using a % pixel-change based assessment (Figures 3C,D). The activity was divided into three types: high, moderate, and inactive states. The high and moderate activities reflect burst swimming (escape behavior) and slower speed foraging swimming respectively (Budick and O'Malley, 2000). The inactive state was set between 0 and 0.03% change as a way to quantify the ‘resting tremor’ as a unique phenotype in the OHDA treated zebrafish larvae. OHDA treatment caused a reduction in the frequency of all states (Figure 3C). Typically, OHDA treatment resulted in a 50–60% reduction in activity state transitions for all three states (Figure 3C). As shown in Figure 3D, all larvae spent the majority of their time in the inactive state, with untreated larvae spending ∼80% of their activity in the inactive state, ∼15% in the moderately active state and ∼5% in the highly active state (Figure 3D). The OHDA treated larvae spent a greater fraction of their time in the inactive state (∼90%) with a concomitant decrease in moderate and high activities (5 and 1% respectively).

### Assessment of behavioral response to cannabinoids and equimolar MEMs

Initial experiments were conducted to determine the effective concentration ranges of each of the five pure, individual cannabinoids. In general, the cannabinoids tested acutely showed a similar effect on baseline larval behavior to that previously profiled for CBD (Achenbach et al., 2018). As the concentration was increased, the normal response to a light to dark transition was abolished. The effective concentration range was considered the concentrations between a no observable effect level (NOE) and a level that had a minimal statistically significant effect on behavior (Table 1). At the concentrations of the cannabinoids tested there was a slight opposition to the 150 µM OHDA induced hypoactivity, however, the effects were not significant.

**TABLE 1:** Cannabinoid dilution series testing results. The effects of purified individual cannabinoids was tested acutely on 120 hpf zebrafish larvae. Also, the pure individual cannabinoids were also evaluated for their ability to reverse OHDA-mediated hypoactivity. L/D = Light/dark startle response.

Chemical	Concentration range	Phenotype	OHDA treatment
Cannabidiol (CBD)	0.25–4 µM	Immediate increase in activity at 2 µM Sedative and abolished L/D at 2 µM	No significant change in activity 0.125–1 µM
Cannabinol (CBN)	0.25–4 µM	Immediate increase in activity at 1 µM Sedative and abolished L/D at 1 µM	No significant change in activity 0.1–1 µM
Cannabichromene (CBC)	0.1–3 µM	Immediate increase in activity at 0.5 µM Sedative and abolished L/D at 0.5 µM	No significant change in activity 0.1–0.5 µM
Cannabidivarin (CBDV)	0.25–4 µM	Immediate increase in activity at 2.5 µM Sedative and abolished L/D at 2.5 µM	No significant change in activity 0.1–0.5 µM
Cannabigerol (CBG)	0.25–3 µM	Immediate increase in activity at 2 µM Sedative and abolished L/D at 2 µM	No significant change in activity 0.25–1 µM

**FIGURE 4:**
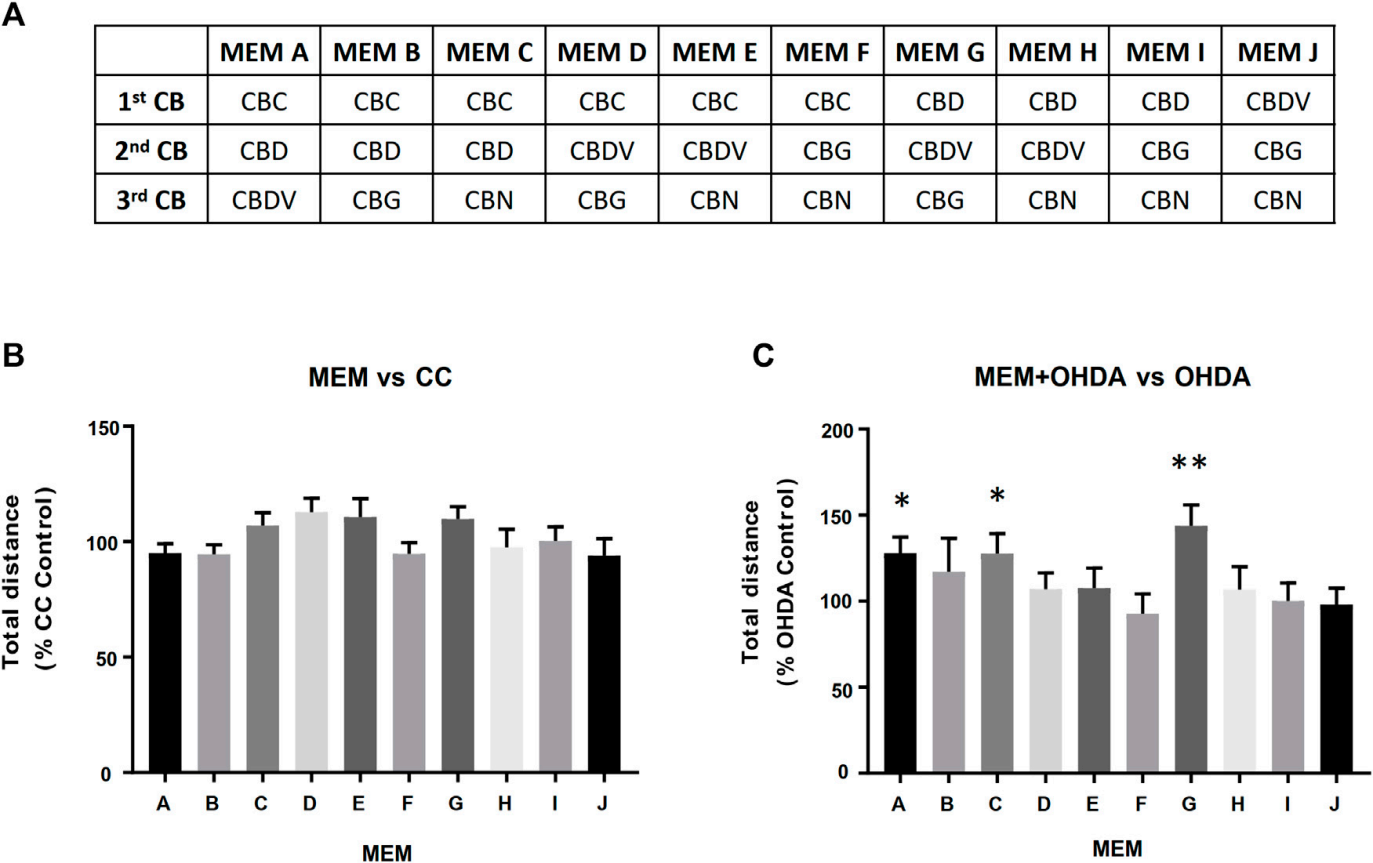
Equimolar Minimum Essential Mixtures (E-MEM) alleviate OHDA mediated hypoactivity. Panel **(A)** Five cannabinoids (CBs) were used to create the 10 possible three component equimolar mixtures. Panels **(B**,**C)** Each of the 500 nM E-MEM (166.7 nM of each cannabinoid) was assessed for its ability modify total distance travelled of **(B)** Carrier Control (CC) or **(C)** 150 µM OHDA. Data is normalized to either CC **(B)** or OHDA **(C)** (100%). MEM + OHDA vs. OHDA, * = *p* < 0.05, **p < 0.01.

In order to assess possible potentiating effects between the cannabinoids, three component, equimolar minimum essential mixtures (E-MEM) of the 5 cannabinoids were prepared (Figure 4A). Three of these E-MEMs; A, C and G showed a significant opposition to the OHDA induced hypoactivity as measured by the total distance travelled (Figure 4C) while not displaying any effect on carrier control larvae (Figure 4B).

**FIGURE 5:**
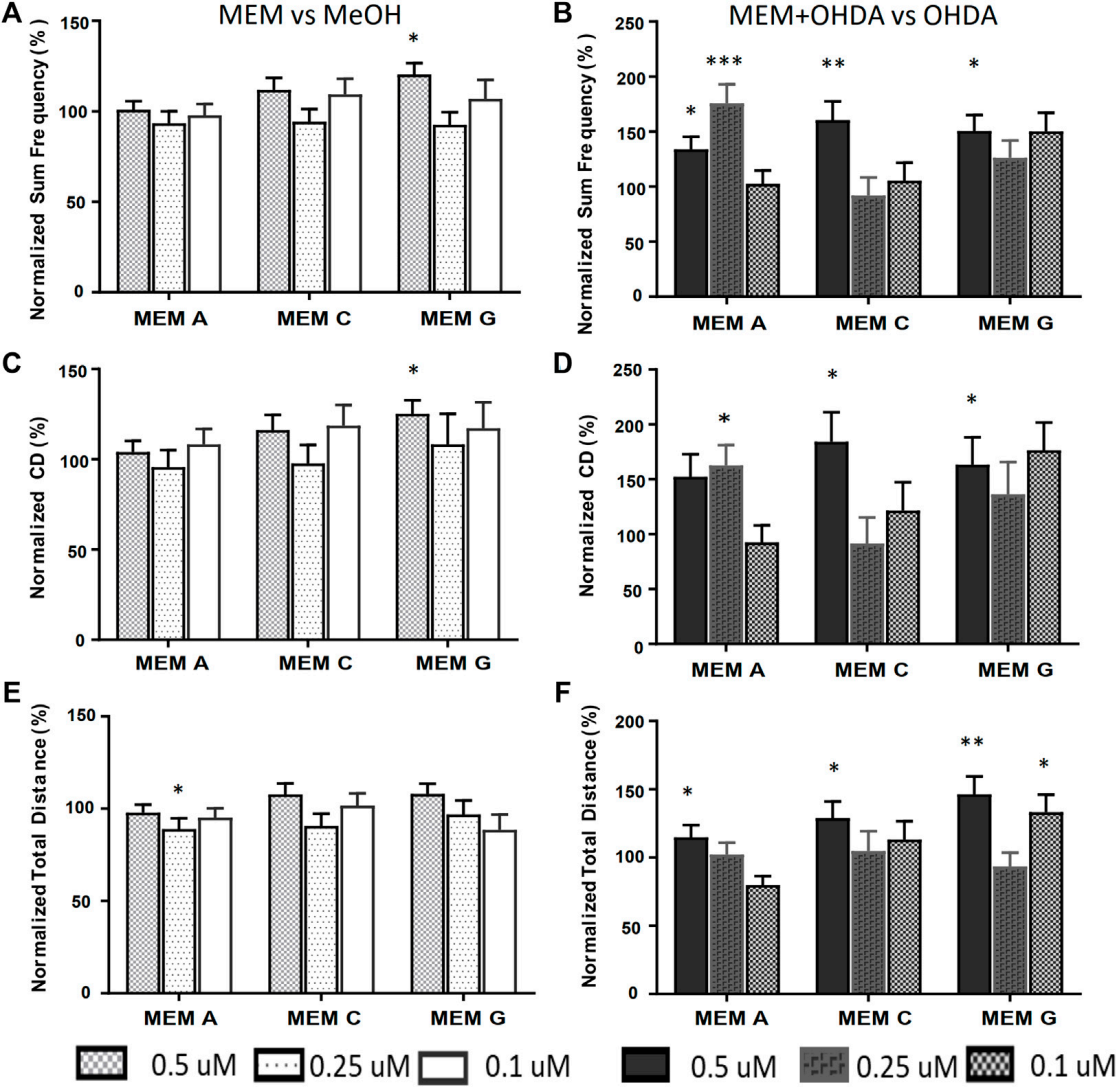
Equimolar MEMs alleviate both OHDA mediated Total Frequency and Cumulative Duration deficits. Activity metrics of Total Frequency **(A,B)**, Cumulative Duration **(C,D)** and Total Distance **(E,F)** for equimolar MEMs at 500, 250 and 100 nM (166.7, 83.3 and 33.3 nM each ingredient respectively). MEM vs. methanol carrier control **(A,C,E)**, and MEM+150 µM OHDA vs. 150 µM OHDA **(B,D,F)**. *p < 0.05, **p < 0.01, ***p < 0.001.

The equimolar MEMs (A, C and G) were further analyzed using the refined activity metrics. At 500 nM all three E-MEMs except E-MEM-C, significantly affected Frequency, Cumulative Duration (CD) and Total distance as measured using refined activity metrics vs OHDA (Figures 5B,D,F). 250 nM dilutions of the E-MEMs abolished these changes, except with relation to Frequency and CD in 250 nM E-MEM A (Figure 5B). No recovery was apparent for the 100 nM E-MEM experiments for Frequency or CD metrics. 500 nM MEM G increased the activity of MeOH treated control larvae as measured by Frequency and CD, (Figures 5A,C), but not total distance (Figure 5E).

### Identification of optimal, defined cannabinoid-ratio minimum essential mixture

**FIGURE 6:**
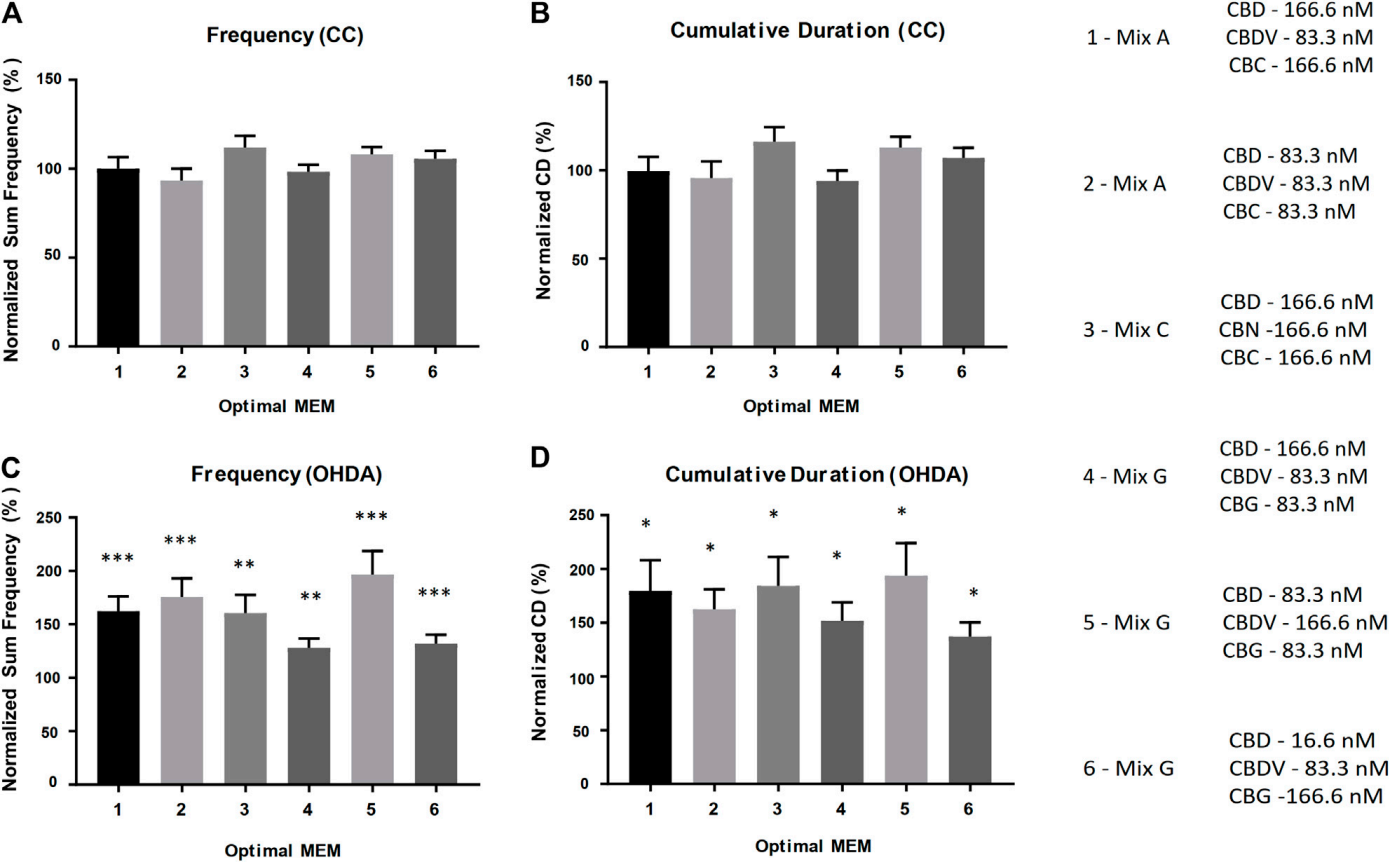
Optimal DCR-MEM ratios alleviate both OHDA mediated frequency and cumulative duration deficits. Activity metrics of Total Frequency **(A–C)** and Cumulative Duration **(B–D)**. Optimal DCR-MEM vs. methanol carrier control **(A,B)**, and Optimal DCR-MEM+150 µM OHDA vs. 150 µM OHDA **(C,D)**. * = *p* < 0.05, ** = *p* < 0.01, *** = *p* < 0.001.

Based on the results from the equimolar mixture experiments, a comprehensive series of experiments were performed where one or two components of each mixture were reduced by 50% (1:2 ratio relative to the equimolar MEM) and/or 90% (1:10 ratio relative to the equimolar MEM) to produce novel molar ratios for further efficacy studies. The defined cannabinoid-ratio minimum essential mixtures (DCR-MEMs) that produced the most significant opposition to the PD-like effects of OHDA will be described herein (Figure 6) and a comprehensive summary of all of the results is included in Table 2. Optimization of all three E-MEMs led to a substantial opposition to PD-like, OHDA mediated changes in Frequency and Cumulative Duration, while having no effect on methanol treated controls (Figure 6). For E-MEM A, the 250 nM equimolar mixture performed as well as the optimized version (a 50% decrease in CBDV from the original 500 nM dilution). None of the MEM C optimized ratios were able to improve upon the 500 nM original dilution without causing increased activity against methanol controls. Several optimized ratios were developed for MEM G that improved upon the original equimolar response to OHDA, while not affecting methanol treated larvae. Specifically, ratio 5 displayed the greatest response in both the Frequency and Cumulative Duration metrics (Figure 6).

**TABLE 2:** Summary of Efficacy of Defined Cannabinoid Ratio MEM activities in the OHDA assay in zebrafish. The concentrations of each of the three cannabinoids tested shown in each field of this table are shown starting with the CBD concentration (labeled in the top row). The CBD concentration is used to divide the results table into three columns. The second cannabinoid and concentration for each ratio result is in the first column of the table and is used to further divide the table into nine sets of nine results, and the last cannabinoid and concentration is above the cell in the row containing the label of the original equimolar ratio formula. Astrices (* = *p* < 0.05, ** = *p* < 0.01, *** = *p* < 0.001) on either side of the “/” represent the level of statistical significance in change in the Total Frequency of Activity State Change metric (right-side)/Cumulative Duration metric (left-side) of zebrafish exposed to the MEM + OHDA versus the OHDA-alone group. Zeroes represent no statistically-significant change in activity, “-” indicates a further reduction (increase in PD-like symptoms, *p* < 0.05)) in activity. N/A = combinations not selected because of the inactivity of their precursors. Bolded cells also showed an MEM dependant increase on methanol treated control larvae.

	166.6 nM CBD			83.3 nM CBD			16.6 nM CBD		
**MIX A**	166.6 nM CBDV	83.3 nM CBDV	16.6 nM CBDV	166.6 nM CBDV	83.3 nM CBDV	16.6 nM CBDV	166.6 nM CBDV	83.3 nM CBDV	16.6 nM CBDV
166.6 nM CBC	*/*	***/*	0/0	*/*	0/0	0/0	*/0	0/0	0/0
83.3 nM CBC	0/0	0/0	0/0	**0/0**	***/*	N/A	0/0	N/A	N/A
16.6 nM CBC	**/0	0/0	**/0	0/0	N/A	N/A	0/0	N/A	0/0
**MIX C**	166.6 nM CBN	83.3 nM CBN	16.6 nM CBN	166.6 nM CBN	83.3 nM CBN	16.6 nM CBN	166.6 nM CBN	83.3 nM CBN	16.6 nM CBN
166.6 nM CBC	**/*	0/0	*/0	0/0	0/0	0/0	**0/0**	0/0	0/0
83.3 nM CBC	0/0	***/*	0/0	**0/0**	0/0	N/A	**/0	N/A	N/A
16.6 nM CBC	0/0	*/0	**/0	0/0	N/A	N/A	**0/0**	N/A	0/0
**MIX G**	166.6 nM CBDV	83.3 nM CBDV	16.6 nM CBDV	166.6 nM CBDV	83.3 nM CBDV	16.6 nM CBDV	166.6 nM CBDV	83.3 nM CBDV	16.6 nM CBDV
166.6 nM CBG	*/*	*/0	0/0	*/*	0/0	0/0	*/*	***/*	*/0
83.3 nM CBG	0/0	**/*	0/-	***/*	0/0	N/A	0/0	N/A	N/A
16.6 nM CBG	**0/0**	0/0	0/0	0/0	N/A	N/A	0/0	N/A	0/*

## Discussion

### Summary of findings: Use of the zebrafish OHDA model to reduce complexity of cannabinoid mixtures that demonstrated efficacy in cell-based MPTP-neuroprotection and dopamine release assays

Anecdotal and Patient Reported Outcomes (PRO) suggest that c Cannabis can alleviate the symptoms of several neurological disorders, including PD. Analysis of clinical meta-data offers mixed evidence in support of medical cannabis. While well tolerated, medical cannabis has been shown to have limited benefit in improving dyskinesia and motor function (Thanabalasingam et al., 2021), although patients did report improvements in sleep quality and quality of life (Urbi et al., 2022). Conversely, cannabis-derived phytocannabinoids have shown a clear neuroprotective effect in rodent models (Prakash and Carter, 2021). While these meta-analyses reveal that medicinal cannabis has therapeutic potential, in order to become a recommended intervention in the treatment of PD, more studies will be required.

However, the use of cannabis as a therapeutic is hampered by a number of factors, including the presence of psychoactive compounds, potential negative chemical interactions, and non-standardized drug delivery methods (inhalation, edibles, dermal). Because there are hundreds of ingredients in cannabis extracts that may have therapeutic potential along with an extremely large number of possible combinations of these constituents, it may be impossible to systematically test every combination in order to identify the optimal mixture of purified components that make cannabis derived compounds good potential therapeutics. Therefore, one approach to identify the essential elements in cannabis is to logically and experimentally reduce the complexity of these mixtures while retaining as much of the original bioactivity as possible. More specifically, one may increase the probability of a positive therapeutic outcome by identifying the top performing mixture at each level of reduced complexity without exhaustively (and blindly) attempting to test every possible variation.

In this paper, we demonstrated the utility of a multitiered approach to identify minimum essential mixtures that are pharmacologically active in cell and animal models of PD. The research presented in this manuscript is predicated on a previously identified patented mixture of cannabis derived compounds (U.S. Patent Number 10,653,640). In our previous work, an *in-silico* database containing the relative percentages (% wt/wt) of the components found in extracts from different varieties of the cannabis plant (Reimann-Philipp et al., 2020) was used to identify a pool of cannabis components with therapeutic potential. The efficacy of >1,000 complex mixtures derived from this original pool of cannabis components were tested using dopaminergic cell models of PD (U.S. Patent Number 10,653,640). Based on this information, the most effective preliminary mixtures were reduced to complex mixtures derived from 10 cannabis-based compounds containing both cannabinoids and terpenes for further testing. We used two cell models to initially reduce the complexity of this patented mixture and to define the most efficacious minimum essential mixtures in the animal model. The MPTP/MPP^+^ assay used in this study models several aspects of PD pathology, including mitochondrial dysfunction and calcium dysregulation, neuronal cytotoxicity resulting from the calcium dysregulation in the mitochondria and the concomitant increase in the production of reactive oxygen and nitrogen species (Cassarino et al., 1997). For this assay we chose Cath.a cells, which are murine neurons from the Locus Coeruleus (LC), one of the earliest sites of PD neurodegeneration (Paredes-Rodriguez et al., 2020). Additionally, because cannabinoids reportedly modulate DA secretion (More and Choi, 2015), we also used dopamine-release assays to measure the ability of these compounds to supplement low dopamine production levels through increased DA secretion. PC12 cells are dopaminergic neuroendocrine cells that have been extensively characterized and are highly amenable to pharmaceutical manipulation (Zhang G. et al., 2019). The cell-based data demonstrated clearly that the cannabinoids provided both neuroprotective and dopamine secretion abilities not seen in the terpene mixtures alone (Figures 1, 2). Because PD has a complex pathology, no single cell line can recapitulate all of the mechanistic details. Thus future work will endeavor to continue to validate the cell line observations in additional animal models, potentially using *ex vivo* cultures of dopaminergic neurons. Importantly, in both of these assays, the cannabinoids demonstrated positive interactions that may be explained by the entourage effect. While more data and modeling are required to determine whether the activity of these combinations represent true synergy (Lederer et al., 2019), the maximal effects seen in the mixtures do represent more than the expected sum of their individual effects. Based on these findings, the terpenes were eliminated from the pool of compounds prior to testing the candidate Minimum Essential Mixtures (MEMs) in the zebrafish model of PD.

A larval zebrafish model was selected to assess the ability of the cannabinoid mixtures to alleviate the movement disorders associated with Parkinson’s disease. When larval zebrafish are exposed to 6-hydroxydopamine (OHDA), there is a dose-dependent effect that leads to the inactivation and eventual death of DA-producing cells of the substantia nigra (Zhang W. et al., 2019). DA-producing neuronal cell loss and associated DA depletion in the striatum are correlated with altered motor behavior and changes in the movement patterns of zebrafish (Feng et al., 2014; Cronin and Grealy, 2017; Benvenutti et al., 2018). Zebrafish larval locomotion is often divided into burst swimming (high velocity) and slow swimming (lower velocity) states (Budick and O'Malley, 2000). Not surprisingly, these disparate behaviors have been mapped to multiple different regions of the brain that are enervated substantially by dopaminergic neurons (Drapeau et al., 2002; McLean and Fetcho, 2004; Severi et al., 2014).

In the current study, we observed a third activity state, characterized by a zero-velocity, tremor-like movement. Attempts to quantify this activity led us to develop advanced analytics of larval behavior, and further evaluation of the etiology of this phenotype is ongoing. The use of the zebrafish OHDA model allowed for a reduction in the complexity and a refinement of the ratios of the original cannabinoid mixtures that demonstrated efficacy in the cell-based assays. MEMs containing CBD and CBDV or CBD and CBC demonstrated the greatest therapeutic potential. These MEMs can now be tested in additional, higher-cost, preclinical model systems.

### Support for evaluating the endocannabinoid system as a drug development target for Parkinson’s disease

While cannabinoids have been suggested as potential agents for treating a spectrum of neurological disorders, including PD, the mechanism by which they exert these actions in unknown. *A priori*, cannabinoids would appear to be promising drugs for targeting the mechanistic pathways that underlie PD with respect to their effects on movement and potentially modifying disease progression through neuroprotective actions. The minor cannabinoids are ligands for a number of receptors, including CB1, CB2, peroxisome proliferator-activated receptors (PPARs), serotonin 5-HT1a receptors, TRPV1 and others (Walsh et al., 2021). These receptors likely play roles in a number of the known multifactorial etiologies of PD, including mitochondrial Ca2+ homeostasis, intracellular Ca2+ signaling, reactive oxygen species, and neuro-inflammatory pathways (More and Choi, 2015). Increasing evidence supports a modulatory role for the ECB system in movement and movement disorders through bi-phasic modulation of dopaminergic, glutamatergic, and GABAergic receptors within ECB retrograde-signaling systems (Catlow and Sanchez-Ramos, 2015; More and Choi, 2015). The human endocannabinoid (ECB) system is therefore an attractive target for the development of novel treatments for neurologic disorders.

Preclinical studies using exogenous cannabinoids have shown their ability to act as neuroprotectants for dopamine (DA)-producing neurons, to reduce oxidative stress and neuroinflammation, and to provide relief from the motor symptoms of PD (More and Choi, 2015; Bandres-Ciga et al., 2020). Loss of dopaminergic neurons in the substantia nigra pars compacta (SNpc) region and their projecting fibers in the striatum are one of the core pathological features of Parkinson’s disease (Gonzalez-Rodriguez et al., 2020). A significant body of literature also demonstrates that cannabinoids can individually restore mitochondrial Ca2+ homeostasis, intracellular calcium signaling, and protect against cytotoxic oxidative stress that would otherwise harm DA-producing neurons (Ryan et al., 2009; Turner et al., 2017; Peres et al., 2018). PD patients in PRO studies reported motor symptom relief from cannabis use; where they reported improvements/decreases in bradykinesia, muscle rigidity, tremor, and dyskinesia in decreasing order of significance (More and Choi, 2015). The data in this study support the idea that ECB-targeting will modify Parkinsonian movement disorders and provides a MEM for further experimentation.

In this work, cell models of PD allowed for the screening of complex mixtures of cannabis-derived ingredients and identified the cannabinoids as those being responsible for the neuroprotective (MPTP/MPP^+^ assays) and dopamine secretory effects. Using the OHDA zebrafish model of Parkinsonian movement disorders, three MEMs containing equal parts of three cannabinoids each were identified that could significantly relieve the OHDA-related motor symptoms. An additional 63 variations using different ratios of the 3 original MEM were also tested, and 22 out of 63 of the defined cannabinoid ratio variations also significantly improved OHDA-related symptoms in zebrafish. Five of these 22 MEMs outperformed the original mixtures and will be further tested in more advanced animal models to develop new therapeutic options for Parkinson’s patients.

## Conclusions

Cannabis has therapeutic promise in PD. However, there is a need to move beyond whole plant extracts and generate safe, reproducible medicines for patients. This paper identified promising minimal essential mixtures of cannabinoids based on a step-wise, strategic approach to reducing the complexity of the plant secondary metabolome. The sequential use of *in silico*, *in vitro,* and medium throughput *in vivo* experimental systems has generated refined, de-risked, mixtures that can now be tested in additional, higher-cost, preclinical model systems of PD.

## Data Availability

The raw data supporting the conclusions of this article will be made available by the authors, without undue reservation.

## Supplementary material

Supplementary Figure S1 Preliminary dilution range testing of cannabinoids in MPTP/MPP^+^ protection assay. The percent protection from MPTP/MPP^+^ induced cytotoxicity was initially measured at 5 concentrations to establish an effective dose range. * = *p* < 0.05. See manuscript for methods.

Supplementary Figure S2 Schematic representation of exposure paradigms for cell culture MPP^+^/MPTP survival and Dopamine release assays, and the 6-OHDA larval zebrafish assay. Test = addition of test compound or mixture. hpf = hours post-fertilization.

## Glossary

ANOVA: Analysis of Variance
Ca: calcium
CaCl2-2H2O: Calcium chloride
CB1: Cannabinoid receptor 1
CB2: Cannabinoid receptor 2
CBC: Cannabichromene
CBD: Cannabidiol
CBDV: Cannabidivarin
CBG: Cannabigerol
CBN: Cannabinol
CC: Carrier Control
DA: Dopamine
DCR-MEM: Defined Cannabinoid-Ratios Minimal Essential Mixture
ECB: Endocannabinoid
E-MEM: Equimolar Minimal Essential Mixture
GABA: Gamma amino Butyric Acid
h: hour
HE3: HEPES buffered E3
Hpf: Hours Post Fertilization
KCl: Potassium chloride
LID: Levodopa Induced Dyskinesia
Lim: Limonene
Lin: Lanalool
MEM: Minimal Essential Mixture
MeOH: Methanol
MgSO4-7H2O: Magnesium hydrate heptahydrate
MPP: 1-methyl-4-phenylpyridinium
MPTP: 1-methyl-4-phenyl-1,2,3,6-tetrahydropyridine
NaCl: Sodium Chloride
Nero: trans nerolidol
OHDA: 6-hydroxydopamine
PD: Parkinson’s disease
Phyt: Phytol
Pin: alpha pinene
PRO: Patient Reported Outcomes
SNpc: Substantia Nigra pars compacta
TH: Tyrosine Hydroxylase
THC: Δ-9 tetrahydrocannabinol
TRPV1: Transient Receptor Potential cation channel subfamily V member 1
