# Supplementary figures and images for: Identification of minimum essential therapeutic mixtures from cannabis plant extracts by screening in cell and animal models of Parkinson’s disease
Source: Front Pharmacol. 2022 Oct 5;13:907579. doi: 10.3389/fphar.2022.907579 (PMC9586206; doi:10.3389/fphar.2022.907579)

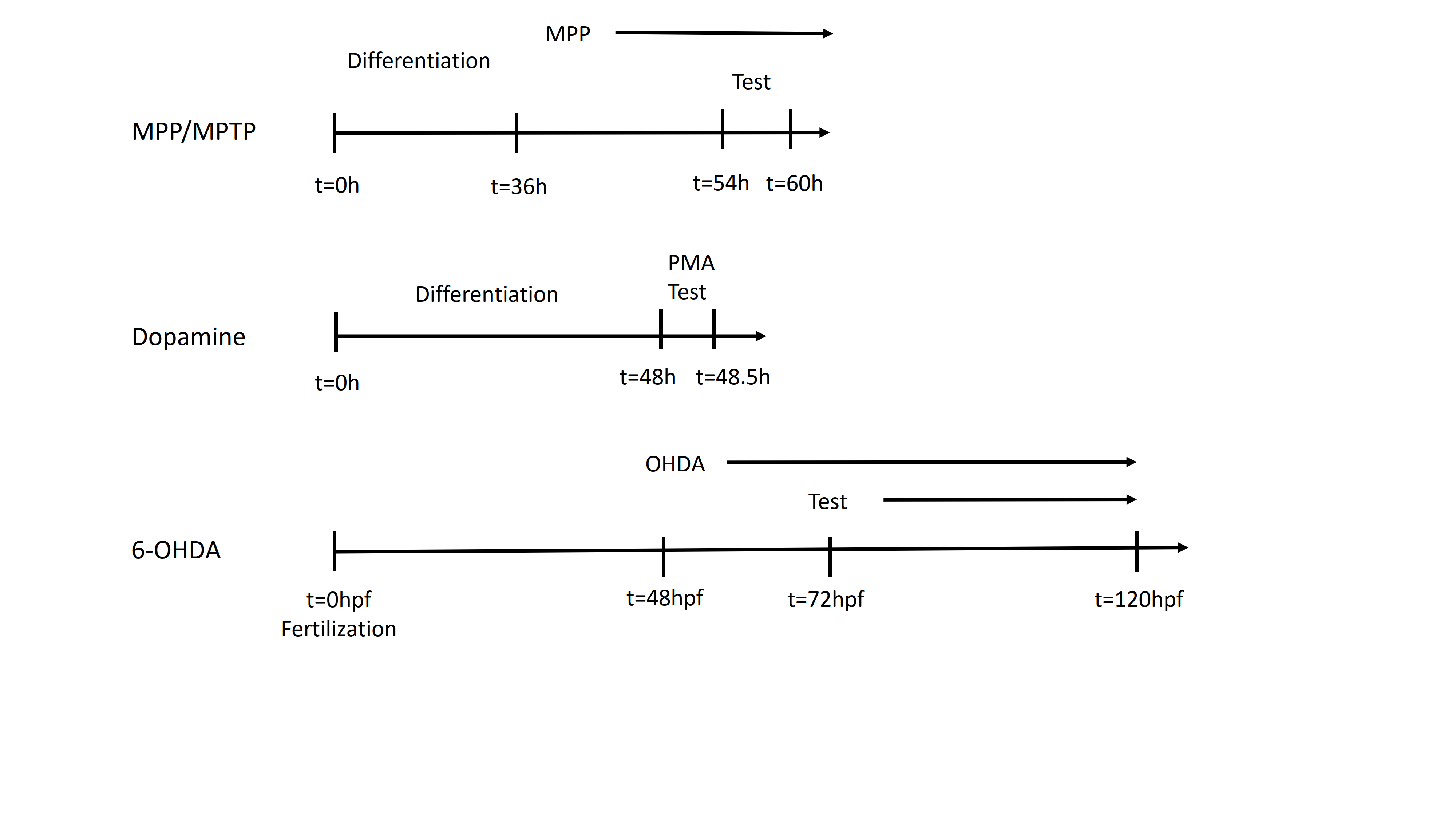

Supplement: Supplementary file 1 [file Image2.TIF]

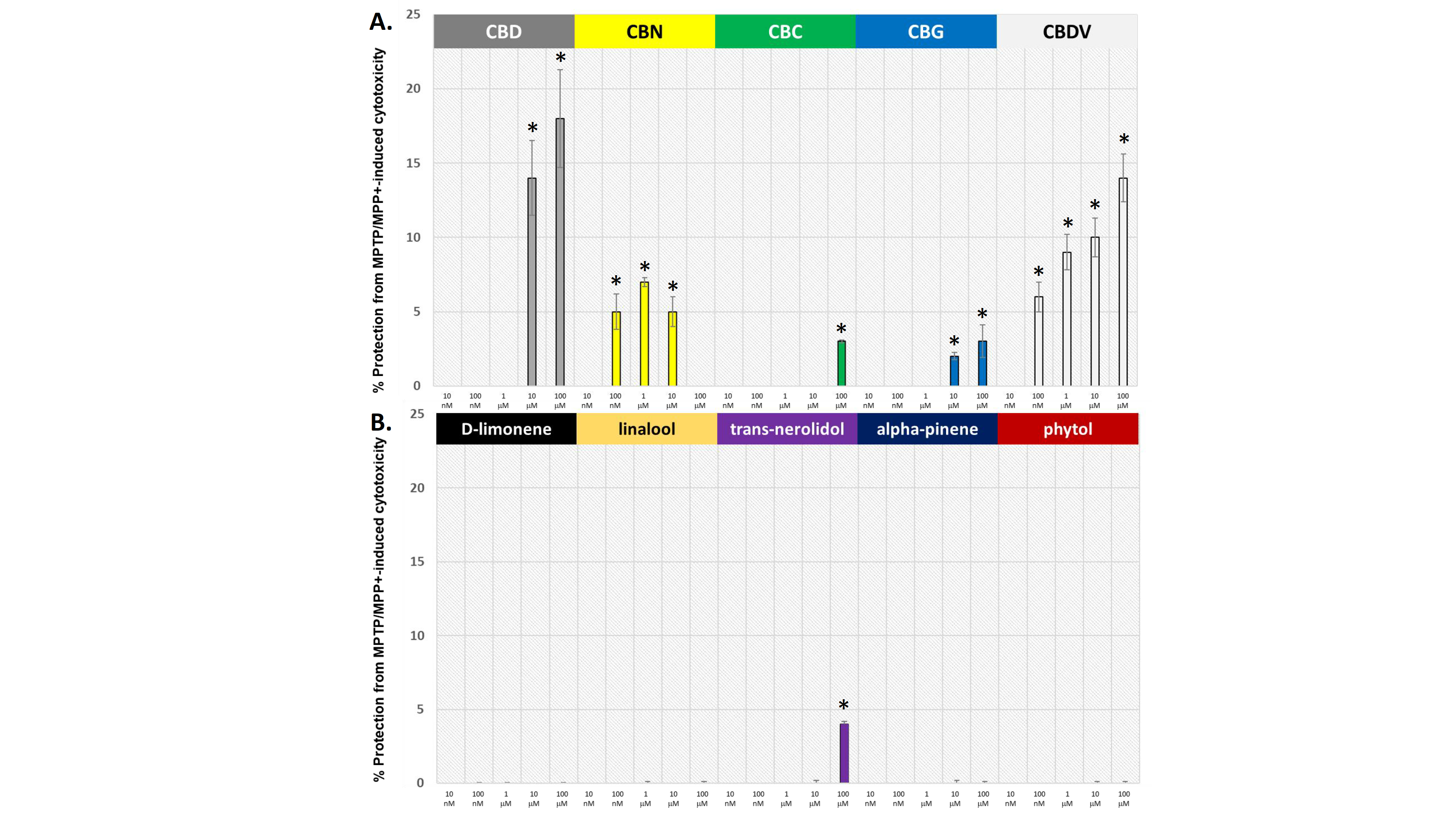

Supplement: Supplementary file 2 [file Image1.TIF]
